# Chitosan-Based Agronanochemicals as a Sustainable Alternative in Crop Protection

**DOI:** 10.3390/molecules25071611

**Published:** 2020-04-01

**Authors:** Farhatun Najat Maluin, Mohd Zobir Hussein

**Affiliations:** Institute of Advanced Technology, Universiti Putra Malaysia, Serdang 43400 UPM, Selangor, Malaysia; farhatunnajat@yahoo.com

**Keywords:** sustainable agriculture, chitosan-based agronanochemicals, crop protection, toxicity, uptake, translocation

## Abstract

The rise in the World’s food demand in line with the increase of the global population has resulted in calls for more research on the production of sustainable food and sustainable agriculture. A natural biopolymer, chitosan, coupled with nanotechnology could offer a sustainable alternative to the use of conventional agrochemicals towards a safer agriculture industry. Here, we review the potential of chitosan-based agronanochemicals as a sustainable alternative in crop protection against pests, diseases as well as plant growth promoters. Such effort offers better alternatives: (1) the existing agricultural active ingredients can be encapsulated into chitosan nanocarriers for the formation of potent biocides against plant pathogens and pests; (2) the controlled release properties and high bioavailability of the nanoformulations help in minimizing the wastage and leaching of the agrochemicals’ active ingredients; (3) the small size, in the nanometer regime, enhances the penetration on the plant cell wall and cuticle, which in turn increases the argochemical uptake; (4) the encapsulation of agrochemicals in chitosan nanocarriers shields the toxic effect of the free agrochemicals on the plant, cells and DNA, thus, minimizing the negative impacts of agrochemical active ingredients on human health and environmental wellness. In addition, this article also briefly reviews the mechanism of action of chitosan against pathogens and the elicitations of plant immunity and defense response activities of chitosan-treated plants.

## 1. Introduction

The increased usage of agrochemicals due to the increase in the World’s food demand urges the need for more research on sustainable agricultural production systems, hence, a heightened drive in agriculture and food security [1,2]. However, the excessive use of agrochemicals represents a significant hindrance in achieving global agriculture security as it poses a negative impact on human health and environmental wellbeing. During the last decade, the global annual agrochemical consumption was approximately 2 million tonnes, where China is the major consumer with 1.8 million tonnes/year, followed by the United States (USA) and Argentina with 500,000 and 236,000 tonnes/year, respectively, compared to only 49,199 tonnes/year used in Malaysia [3]. Moreover, the global usage of agrochemicals has been estimated to rise by 3.5 million tonnes in 2020 [4]. The term agrochemicals covers a wide range of substances, including fungicides, insecticides, herbicides, rodenticides, fertilizers, plant growth stimulants, etc. [5]. In general, agrochemicals are used in crop management to enhance crop productivity and yield, reduce crop losses, combat plant-related diseases and increase food quality [6]. Alongside their benefits, agrochemicals are also known for their toxic properties, hence posing a threat to living organisms in the soil and rivers due to losses during their application via degradation, volatilization, photolysis and leaching. Furthermore, only 0.1% of the applied agrochemicals are delivered to the particular target site and act effectively against the target organism (i.e., insect, bacteria, fungi, or virus) [7].

Nano-enabled agrochemicals, also referred to as agronanochemicals, could hold the key in the development of integrated management of pests and diseases, as they offer controlled release of active ingredients and site-specific delivery, thus, increasing their efficacy and efficiency [8,9]. They provide a sustainable alternative for pest and disease management in crops [10]. Apart from that, agronanochemicals can surmount the environmental contamination issues arising from the excessive usage of conventional agrochemicals [11]. Moreover, agronanochemicals can lower the toxicity of agrochemicals, enhance agrochemical uptake, improve solubility and stability, as well as minimize volatilization, leaching and run-off of agrochemicals that can cause environmental and health concerns [12].

## 2. Chitosan-Based Agronanochemicals

According to the United Stated (USA) Food and Drug Association (FDA), chitosan is established as a non-toxic, biodegradable, and biocompatible compound [13]. It offers fascinating properties; antimicrobial, antiviral, antifungal, antioxidant, anti-inflammatory, bio-adhesion, adsorption enhancer, etc. [14]. Chitosan is soluble at acidic pHs due to the protonation of its amino group. It is derived from chitin via chemical deacetylation under alkaline conditions, where chitin is the second most abundant natural biopolymer and can be found in the shell of crustaceans, insect cuticles and fungal cell walls [15]. Besides, the production of chitosan is one of the ways to utilize the bio-waste that comes from the crustacean production industries, where its global production are approximately 6–8 million tonnes/year with 1.5 million being produced by Southeast Asian countries [16]. This is an effort towards achieving a “zero-waste” food industry, hence benefiting to both the economy and the environment [17]. Nevertheless, it is worth noting that the production of 1 kg of chitosan consumes over than 1 tonne of water. Therefore, the utilization of chitosan-based agronanochemicals as a sustainable alternative in crops management has raised a debate among researchers. However, we believe that the advantages of chitosan production to convert waste to wealth, together with the positive outcomes of chitosan nanoformulations in crops management; especially the synergistic effect, toxic-protection abilities, minimization of agrochemical leaching and runoff to the soil and water body, high potency, high efficiency etc., outweigh the need for a huge amount of water during the production of chitosan. The controlled release formulation and high bioavailability could overcome environmental and health issues such as run-off and accumulation of agrochemicals, as well as helping in reducing the labor cost in the agricultural industry. The low toxicity properties make them harmless to the farmers and the person who will be applying it. Again, all in all, the benefits of chitosan-based agronanochemicals outweigh the huge water consumption required for the production of chitosan and therefore it is a way forward, especially for crop management.

In agriculture, chitosan nanoparticles by themselves can act as growth enhancers and potent antimicrobial agents against pathogenic fungi and bacteria [18]. Alternatively, they can also act as a nanocarriers for existing agrochemicals, which hence are referred to as chitosan-based agronanochemicals [19,20,21]. The nanocarrier system enables the agriculturally active ingredient to be encapsulated via ionic or covalent inter/intramolecular bonds or entrapped in a polymeric matrix of chitosan to develop an effective nanodelivery system formulation [21]. Chitosan-based agronanochemicals can be prepared using several methods, including ionic gelation, emulsion cross-linking, spray drying, precipitation, reverse micellar and sieving methods [22]. Out of these methods, the sieving method is the simplest and direct method. However, the method has been reported to produce nanoparticles of irregular shape and size. On the other hand, the ionic gelation method is the subject of intense research in the formulation of chitosan nanoparticulate systems due to its simplicity and relatively cheap cost. The method does not require many chemicals, hence reducing the possible toxic side effects. It also employs the use of polyanions with a negative charge (e.g., tripolyphosphate) to bond with the positive charge of the protonated amino group of chitosan under acidic conditions. The emulsion cross-linking method produces stable nanoparticulate systems, however, the process is quite tedious and requires crosslinking agents such as glutaraldehyde, formaldehyde, alginate, etc. which might cause complications due to their incompatibility with the active ingredients. The resulting particle size mainly depends on the emulsion droplet size which in turn depends on the crosslinking degree, molecular weight of the chitosan, surfactant type, and the speed of stirring.

A thermodynamically stable, small particle size and uniform size distribution chitosan nanoparticulate system can be achieved by a reverse micellar method. The method requires a surfactant solution such as cetyl trimethylammonium bromide, an organic solvent, and a crosslinking agent, hence, this method is not desirable due to its laborious and expensive procedures despite the advantages. Precipitation methods involves blowing a chitosan solution using a compressed air nozzle, thus forming coacervate nanoparticles. The downside of this method is that the resulting nanoparticles are not stable, having irregular shapes and low mechanical strength. Spray drying methods have been widely used in the production of dry granules, powders and pellet forms of chitosan. The methods employ the consecutive addition of active ingredients and crosslinking agent to the chitosan solution dissolved in acetic acid. The solution then undergoes an evaporation process under a hot air stream to form the desired nanoparticles.

Nanoformulations aim to enhance the benefits of chitosan and agrochemicals while simultaneously reducing the adverse outcomes. Due to its amphiphilic properties, the encapsulation of chitosan could overcome the poor solubility of many agrochemicals in water, providing an alternative use of inert chemicals in conventional agrochemicals, hence, subsequently reducing theirs toxicity level [19]. The bioadhesive properties in chitosan provide excellent protection to the encapsulated agrochemicals, thus, increasing the stability and bioavailability in the plant [23].

### 2.1. Controlled Release Formulations

Chitosan-based agronanochemicals exhibit highly controlled release behavior that subsequently increases its bioavailability with high circulation and retention time in the plant tissue (higher half-lives, t_1/2_). Thus, the controlled release of active ingredients in agrochemicals aims to address the problems associated with the excessive usage of agrochemicals by reducing the quantiies and frequency application in the field. The agrochemical release from the chitosan matrix can be triggered by two types of stimuli: (1) biotic stress, such as the presence of plant pathogens (fungi and bacteria), nematodes, insects, pest and weeds; and (2) abiotic stress factors, such as pH, temperature, salinity, flooding, drought and other environmental factors [7,24].

**Figure 1:**
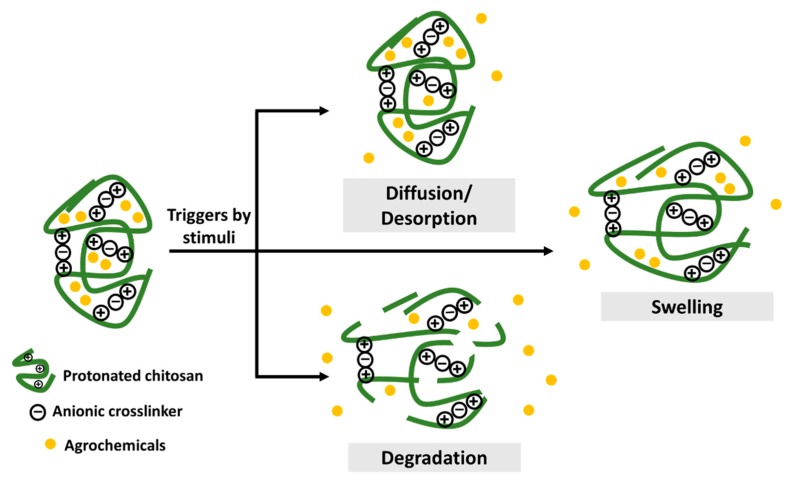
The release mechanism of active agents from chitosan-based agronanochemicals (reproduced based on [21] and [33]).

The release mechanism upon stimulus-response is through pore diffusion, surface desorption, capsule swelling and degradation, as illustrated in Figure 1 [7,25]. The diffusion-controlled mechanism relies on a diffusion rate gradient while the surface desorption refers to the active ingredient adsorbed on the surface of the nanoformulation. Upon hydration, the release of agrochemicals depends on the swelling of the chitosan capsule. Moreover, enzymatic reactions or other environmental factors might result in the rupture or degradation of the capsule matrix. Hence, the controlled release based on the stimuli response in nanoformulations enables the release of the agrochemicals effectively and efficiently at the target site of interest. A pH-dependent release of Cu was observed upon its encapsulation into chitosan nanoparticles, in which the decrease from pH 3 to pH 1 leads to the increased release of Cu from 21.5% to 44.1%, respectively [26]. This is due to the protonation of the chitosan’s amino group. At higher pH of 6 and 7, a drastic decrease of Cu release was observed (6.1% and 4.9%, respectively), due to the deprotonation of the chitosan’s amino group. Moreover, the sustained release of Cu for up to 96 h was obtained at pH 4.5. A stimulus-response release mechanism was observed for chitosan-Zn nanoparticles, in which the Zn release was mainly due to the stomatal uptake, followed by diffusion and swelling of polymers upon water penetration [27]. The slightly acidic environment of the intracellular medium is also reported to be able to help release Zn from chitosan nanoparticles. Chitosan-hexaconazole nanoparticles and chitosan-dazomet nanoparticles demonstrated diffusion-controlled release of the fungicides at pH 5.5 with half release times (t_1/2_) of 42 and 11 h, respectively [28,29]. Moreover, the co-release of hexaconazole and dazomet from the chitosan-hexaconazole-dazomet nanoparticles prolonged the release time with t_1/2_ of 53 and 15 h, respectively [30]. The diffusion-controlled release of methomyl at pH 6.0 with t_1/2_ of 36–70 h has been obtained by its encapsulation into carboxymethyl chitosan and azidobenzaldehyde [31]. The release of the insecticide acetamiprid from nanocapsules of chitosan-alginate was reported to be pH-dependent, in which half of the acetamiprid was released after 36 h at pH 7.0 and 4.0 compared to only 24 h needed to release the same amount at pH 10 [32].

### 2.2. Plant Growth Promoter

The use of nanoformulations of chitosan itself as a plant growth promoter has been extensively researched. The protonated chitosan, rich in positive charges, shows increased affinity towards plant cell membranes, resulting in enhanced reactivity in the plant system. Also, 9–10% nitrogen, which is the main component of chitosan, serves as a macronutrient in a plant [22]. Alternatively, chitosan can be incorporated with plant macronutrients (nitrogen [N], phosphorus [P], potassium [K], magnesium [Mg], calcium [Ca] and sulfur [S]) and micronutrient (copper [Cu], manganese [Mn], nickel [Ni], zinc [Zn], boron [B], iron [Fe] and chlorine [Cl]). The summary of some of the recent works on the use of nanochitosan and macro/micronutrient nanocarriers as a plant growth promoter are listed in Table 1. As shown in Table 1, chitosan nanoformulations have been widely used as an alternative method in seed treatment to promote seed germination and increase biomass accumulation. Moreover, chitosan nanoformulations have also been used as growth promoters by enhancing the nutrient uptake, chlorophyll content and photosynthesis rate. For example, the leaves of Robusta coffee seedlings were sprayed with a high molecular weight chitosan oligomer and nanochitosan at three different average HRTEM diameter sizes: small (420 nm), medium (750 nm), and large (970 nm) [34]. The results indicated that nanochitosan at all sizes exhibited better nutrient uptake (i.e., N, K, P, Ca, and Mg) than chitosan oligomer. The effect of size on the nutrient uptake was found to be not significant. On the other hand, an effect of size can be observed in the chlorophyll content and photosynthesis rate. The amount of chlorophyll content was enhanced up to 61%, 81%, and 61% for small, medium and large sizes of nanochitosan, respectively. Also, the photosynthesis rate was improved up to 29%, 59%, and 72% for small, medium, and large nanochitosan, respectively. The treated seedlings at all sizes of nanochitosan also exhibited better vegetative growth compared to the seedlings treated with chitosan oligomer and the untreated seedlings. In another work, the supplementation with nanochitosan improved the tolerance of bean seedlings to abiotic stress (salinity stress) [35]. Moreover, chitosan-polymethacrylic acid-NPK nanoparticles have been formulated and used for wheat plants [36]. The effectiveness of the nanoformulations was compared with the bulk NPK conventional fertilizer. Upon application of 500 mg/mL of N, 60 mg/mL of P and 400 mg/mL of K of the nanoformulations, the plant height, main spike weight, crop yield and harvest index recorded are 41.29 cm, 0.178 g, 6.95 g/plant, and 26.94, respectively. At the same dosage, the bulk NPK recorded plant heights, main spike weights, crop yields and harvest indexes of 38.85 cm, 0.136 g, 6.13 g/plant, and 21.64, respectively, hence showing the high potential of nanoformulations as plant growth and crop yield enhancers of wheat. The effect of bulk chitosan, copper sulfate (CuSO_4_) and chitosan-Cu nanoparticles on the seedling growth of maize also was investigated [37]. A significant effect of the nanoformulations on seedling growth, total protein content and α-amylase and protease activity compared to the bulk chitosan was observed. It was hypothesized that the nanoformulations might enable seed penetration and subsequently improve the metabolism of the seed, presumably, bulk chitosan could develop a film coating on the seed surface, thus, preventing their access to water and nutrients.

Plant growth regulators can be encapsulated into chitosan nanocarriers for the development of an effective nanodelivery system of hormones in a slow-release manner and with high bioavailability. Plant growth regulators, also known as plant hormones, such as gibberellins, auxins, abscisic acid, cytokinin and ethylene are chemical substances responsible for regulating plant growth and plant cell development. Chitosan-gibberellic acid nanoparticles exhibited a 37% and 82% increase of root development and leaf area in French bean, respectively, compared to the free hormone, gibberellic acid [38]. Apart from that, more lateral roots were formed upon supplementation of chitosan-γ-polyglutamic acid-gibberellic acid nanoparticles on French bean seedlings compared to the free hormone [39], hence highlighting the benefits of the nanoparticulate systems. Chickpea seeds treated with chitosan-thiamine nanoparticles exhibited a higher germination percentage with 90% compared to the mixture of chitosan-thiamine and control (water) with 84% and 75%, respectively [40]. The seedlings treated with the nanoparticulate system also exhibited more defense enzymes and 10-fold higher auxin levels compared to the untreated seedlings.

### 2.3. Biocides Against Plant Pathogens and Pests

Chitosan with or without the incorporation of macronutrients can act as an alternative sustainable potent biocidal agent against pathogenic fungi, viruses and bacteria. A summary of some of the recent works on the use of nanochitosan and its incorporation in plant management is provided in Table 2. As shown in the summary, chitosan with or without the incorporation of other active agents exhibited good potential as a sustainable alternative to the use of conventional fungicides against *Fusarium* head blight and wilt disease in wheat and chickpea, post-flowering stalk rot in maize, blast leaf in rice, blast disease in finger millet and leaf spot in maize, among others.

The nanoformulation of chitosan incorporated with polyacrylic acid offers excellent potential in managing attack of common pests like cotton aphid and beetles in soybean cultivation [41]. Several studies have also revealed the ability of chitosan nanoformulations to boost the plant defense mechanism by eliciting the defense enzyme activities upon its application (the details will be discussed later). In addition, in vitro evaluation of oleoyl-chitosan nanoformulation revealed several chitosan-sensitive fungi with significant antifungal effects, such as *Alternaria tenuissima*, *Nigrospora sphaerica*, *Nigrospora oryzae*, *Botryosphaeria dothidea*, while *Fusarium culmorum* and *Gibberella zeae* can be classified as chitosan-resistant fungi [42]. Moreover, chitosan-Cu nanoparticles-treated in vitro plates effectively inhibited the mycelial growth and spore germination of *Alternaria alternata* (90%), *Macrophomina phaseolina* (63%), and *Rhizoctonia solani* (60%) [25]. Chitosan-polyacrylic acid nanoparticles were found to be fungi-sensitive with a decrease of inhibition percentage as follows: *Aspergillus flavus* (60%), *Fusarium oxysporum* (41%), *Fusarium solani* (40%), *Aspergillus terreus* (40%), *Alternaria tenuis* (40%), *Aspergillus niger* (37%), and *Sclerotium rolfsii* [41]. The antimicrobial activity of nano-chitosan compared to its bulk counterpart on *Pyricularia grisea*, *Alternaria solani* and *Fusarium oxysporum* was investigated [43]. The nanochitosan exhibited higher percentage inhibition of the mycelia growth compared to bulk chitosan. It was reported that the small size, high permeable nature and higher zeta potential of nanochitosan make it more stable and effective against the tested fungal pathogens.

The inhibitory effect of bulk chitosan (BCS), chitosan nanoparticles (CSNps) and chitosan nanoparticles added with ethanolic blueberry extract (CSNps-EBE) on *A. alternata* was observed, where the trend was as follows: CSNps-EBE (83.3%), CSNPs (83.1%) > BCS (6.0%) [44]. Their inhibitory effect on *C. gloeosporioides* follows the trend: chitosan nanoparticles-methanol nanche extract (79.6%) > CSNps (57.0%) > BCS (9.4%). In another work, Kheiri et al. employed three different molecular weights (MW) of chitosan (i.e., low MW of 161 kDa, medium MW of 300 kDa, and high MW of 810 kDa) for the formation of nanoparticulate systems [45]. The resulting nanoparticles exhibit lower zeta potential and a bigger average size with the increase of the molecular weight and thus, subsequently, resulted in lower antifungal activity on *Fusarium graminearum* (in vitro). Low MW of chitosan nanoparticles exhibit 2-fold higher antifungal activity compared to their nanoparticles of medium and high MW chitosan. This is due to the higher charge (more stability) and smaller size (easier cell penetration) of low MW chitosan nanoparticles.

In addition, the chitosan nanodelivery system was loaded with agrochemicals as the active agent, for the formation of chitosan-agrochemical nanoparticles that offer controlled release properties with high efficacy and potency, as the active ingredient can reach the target cell or plant parts more effectively within a defined time [12]. Some of the recent works on chitosan-agrochemical nanoparticles are listed in Table 3. The crucial parameters in the design and preparation of chitosan-agrochemicals nanoparticles include loading content of active agent, encapsulation efficiency of active agent, the release profile of active agent, their particle size and morphology. There are several works reporting on the design and preparation of these nanoformulations by focusing on these parameters. Nanocarrier system of herbicides (diuron) as a photosynthetic inhibitor for the weed control was developed by crosslinking carboxymethyl chitosan and 2-nitro benzyl (140 nm, average HRTEM diameter size). The nanoformulations were developed with a photo-controlled release mechanism [46]. In another work, a smart formulation of chitosan-alginate nanocapsules (30–40 nm diameter size of HRTEM) was developed for the controlled release of acetamiprid, in which the controlled release properties was achieved at three different pHs, where a 50% insecticide release was found after 24 h at pH 10 and after 24 h at pH 7 and 4, compared to only about 6 h for the conventional insecticide release at all pHs [32]. Carboxymethyl chitosan incorporating ricinoleic acid was developed for a 200–500 nm (hydrodynamic size) nanoemulsion of azadirachtin. The nanoformulations enhanced the solubility and stability with a slow and stable release of the insecticides [47].

## 3. The Mechanism of Actions of Chitosan Against the Pathogens

The antimicrobial action of chitosan on pathogens (bacteria, fungi and virus) relies on several mechanisms: (1) the positive charge of the protonated chitosan enables electrostatic interactions with the negative charge of the pathogen surface; (2) the cell damage and leakage of the pathogen, hence increases its membrane permeability and subsequently results in cell death [65]; (3) chitosan then chelates the essential elements (including metal ions, minerals and nutrients) for the growth of pathogens, thus, preventing the normal growth of pathogens; (4) DNA/RNA interaction of pathogens with the penetrated chitosan leads to the inhibition of the mRNA syncretization and pathogen reproduction; and lastly, (5) the deposition of chitosan on the microbial surface of pathogens forms a barrier to extracellular transport of the essential nutrients and metabolites from entering the cell, hence, inhibits the normal growth of pathogens [70,71,72].

The efficiency of the mechanism in action can be enhanced by the small size of the chitosan nanoformulations due to their high surface area that comes in contact with the pathogens. The small size also can enhance the uptake and increase of the penetrated and permeated chitosan on the thick coat of seeds, plant tissues, as well as the cell membranes of pathogens, hence resulting in better elicitations of plant immunity and defense response activities.

Chitosan also offers plant immunity and defense-eliciting properties by inducing the defense-related enzyme such as phenylalanine ammonia-lyase (PAL), polyphenol-oxidase, catalase and peroxidase [73]. PAL is an enzyme that helps in catalyzation of L-phenylalanine to trans-cinnamic acid and ammonium, in which the conversion can be regarded as a critical step in inducing the metabolism in a plant [74]. The reduction in gray mold incidence in pre- and post-harvest with a 2-fold increase of PAL upon treatment with 1% chitosan on grapes has been reported [75]. Similarly, rice and wheat treated with chitosan induced PAL production and reduced disease incidence [76]. The enzyme polyphenol oxidase helps to catalyze the phenolic substances to lignin biosynthesis. Hence, the increase of these enzymes can be interpreted as an increase in lignin formation that contributes to the building up of cell wall structure, hence, forming a barrier for the penetration of pathogens [77].

Catalase is an antioxidant enzyme involved in the decomposition of hydrogen peroxide (H_2_O_2_) to water (H_2_O) and oxygen (O_2_). The enzymes protect the plant cell from oxidative damage by reactive oxygen species (ROS). Seeds primed with chitosan have shown increased catalase activity while at the same time, accelerate germination rated and enhanced tolerance to temperature stress [78]. Plant peroxidases are enzymes that can be found in lignin biosynthesis and exposure to biotic and abiotic stress. The process contributes to the production of toxic ROS. Hence, while catalase protects the plant cells from ROS, the release of ROS is lethal to the pathogen [79]. Correspondingly, high inhibition of spore germination and mycelial growth of *Physalospora piricola* and *Alternaria kikuchiana* with increased peroxidase activity in chitosan-treated pear has been reported [80]. A significantly high peroxidase-gene expression was observed in peaches treated with chitosan compared to the untreated ones [81].

Previous studies have also reported on the accumulation of defense-related secondary metabolites, including phytoalexins, phenolic compounds, lignin and callose in plants treated with chitosan [82,83]. Phytoalexins are toxins that have antimicrobial and antioxidant properties produced upon infection by pathogens, the results of metabolites’ reactions against the disease. Interestingly, plants incorporated with chitosan were also found to be able to elicit phytoalexins. It was first reported in 1980, where at the application of 0.9 μg/mL, chitosan significantly induced phytoalexins and provided resistance to pea plants against *F. solani* attack [84]. Further evidence on the accumulation of phytoalexins upon the treatment of chitosan was reported by Trotel et al., in which a 20% increase of phytoalexins was observed in the grapevine leaves treated at 200 μg/mL chitosan [85]. Awadalla and Mahmoud introduced a new chitosan derivative (carboxymethyl chitosan) as a tool in stimulating phytoalexins and inducing the *Fusarium* wilt resistance in cotton seeds [86]. Plant phenolic compounds play an essential part in providing plant resistance against pathogenic infections by producing lignin, signal compounds (such as flavonoids and salicylic acid), and defense response chemicals (such as phytoalexins and tannins). An elevation of the total phenolic compound level was observed after 60 h of treatment by chitosan in grapes [75]. At 50 and 200 mg/mL of chitosan oligosaccharides, treated oregano showed a significantly upregulated polyphenols content (by 38% and 29%, respectively) [87]. Callose is a polysaccharide found in plant cell walls. Its roles include regulating the permeability plasmodesmata, phragmoplast formation and phloem pores as acts of plant cell protection against abiotic and biotic environmental stimuli [25,88]. The abilities of the chitosan-treated plant in eliciting callose formation in response to pathogenic attacks have been reported in several works [89,90,91].

Moreover, upon chitosan treatment, plants exhibit pathogenesis-related proteins, including chitinase and β-1,3-glucanase [70,92]. In response to pathogen attack, plants induce proteins and peptides with antimicrobial properties to protect themselves. Interestingly, chitosan also can act as an elicitor in inducing the pathogenesis-related proteins and improve the plant resistance against pathogens. Chitinase and β-1,3-glucanase can act as a catalytic converter in the hydrolysis of chitin and β-D-glucans that can be found in the fungal cell wall and insect exoskeletons. Hence consequently, it degrades the fungal cell wall and stops the fungal growth on the host plant [70,93].

## 4. Uptake, Translocation and Transportation of Agronanochemicals in Plant

The uptake, translocation and transportation of agrochemicals in crops, particularly fungicides, play an important role in their effectiveness in combating fungal infections, in which, the failure in the delivery of active fungicide constituents to the target site of the pathogenic fungus might be the reason for the ineffectiveness of the disease control. The uptake efficiency via leaves and roots also could hold the key is the effectiveness of nanoparticles on the metabolic functions and growth of plants. Chitosan was reported able to easily penetrate plant surfaces (i.e., foliar, stem and root) [21]. Moreover, chitosan-based agronanochemical systems help facilitate the uptake and penetration of active agents through to the cell membrane, thus, increasing the bioavailability of active agents inside the plant tissues.

**Figure 2:**
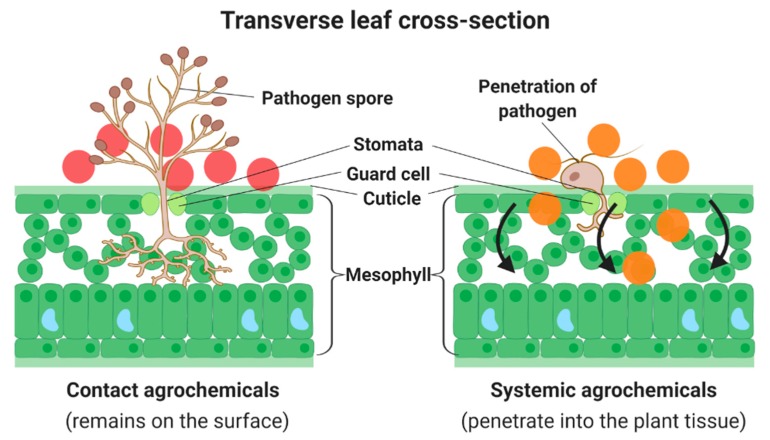
Translocation of foliar-applied agrochemicals.

Agrochemicals can be classified into two main categories—systemic and contact—as shown in Figure 2. Foliar application of systemic agrochemicals is by absorption where the chemicals can penetrate the cuticle leaf and move into the plant tissue via the phloem. Systemic agrochemicals are curative and eradicative treatments, as they can kill the pathogens that may have penetrated in the plant tissue. They also help to halt pathogens infections from spreading throughout the plant. Examples of systemic agrochemicals are benzimidazole, hexaconazole, avermectin, azoxystrobin, pyraclostrobin, etc. On the contrary, contact agrochemicals are adsorbed and remain on the surface of the applied leaf. Hence, contact agrochemicals are known as a preventive treatment as they kill the pathogenic spores before the mycelia can develop and grow inside the plant [94]. However, the use of contact agrochemicals such as copper, sulfur and fludioxonil is no longer useful after the plant has been infected [95].

**Figure 3:**
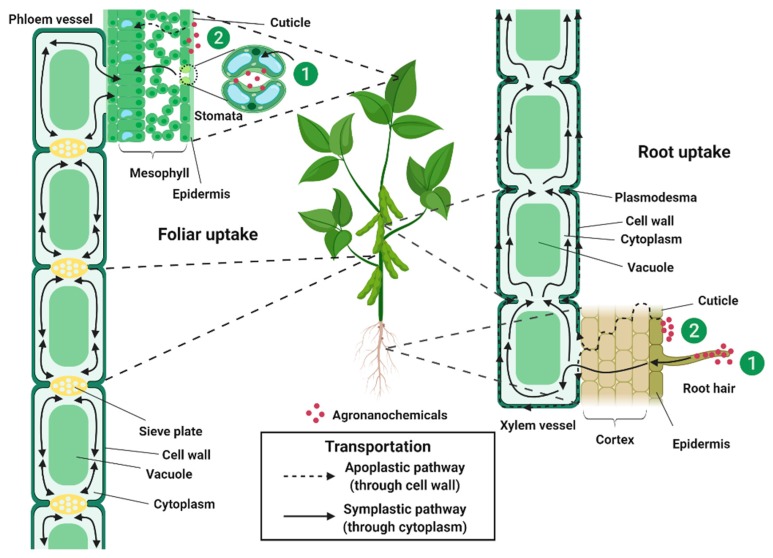
Illustration of an important role of chitosan during uptake, translocation, and transportation of agronanochemicals (reproduced based on [98] and [107]).

Therefore, to develop an effective agronanochemicals delivery system, systemic agrochemicals are usually chosen as the active ingredient (Table 3) where it was hypothesized that the penetration of agronanochemicals into the plant cell could occur through carrier protein binding via endocytosis, ion channels and aquaporin [96]. The uptake of agronanochemicals can be divided into foliar and root exposure (Figure 3). In the foliar uptake, agronanochemicals can be translocated into the plant tissue via: (1) a stomatal pathway or (2) a cuticular pathway [97,98]. Due to their small size, agronanochemicals can easily penetrate the leaf tissue through the stomata openings with the typical stomatal aperture size of about 3–10 μm width and 25 μm length [99]. The diffusion of agronanochemicals into the cuticle pores is usually limited due to the petite size of the cuticle pores (0.6–4.8 nm) [100,101].

There are two possible pathways of the movement of agronanochemicals from soil to root tissues: (1) root diffusion and (2) cuticular pathway. Root diffusion relies on the concentration gradient between the root and soil, which allows the movement from high to low concentrations parts. The uptake of agronanochemicals occurs through the cell wall barrier where the pore diameter of the plant cell wall measured using various techniques has been reported to be generally in the range of 5–50 nm [36,102,103]. However, some other works have also reported the possibility of pore enlargement upon interaction with the agronanochemicals, which in turn increases their uptake [96,103].

The penetrated agronanochemicals are then translocated and transported to the other parts of the plant via phloem and/or xylem, hence, referred to as systemic phloem and/or systemic xylem, respectively [104]. The movement of systemic phloem upon foliar application follows the symplastic pathway (through cytoplasm) and is bidirectional, which means the movement is in two directions, downward (shoot to root) and upward (root to shoot). The movement of systemic xylem upon foliar application follows the symplastic pathway (through cytoplasm) and apoplastic pathway (through cell wall) and is unidirectional, i.e., the upward direction only. Agrochemicals with the ability to move upward and downward through xylem and phloem are called truly systemic agrochemicals, and some examples are harpin, acibenzolar-S-methyl, fosetyl-aluminum, etc. [105]. Interestingly, the movement of chitosan-NPK nanoparticles (a mixture of nanochitosan-N (50 nm), nanochitosan-P [68 nm] and nanochitosan-K (45 nm)) by foliar application on wheat have shown that the nanoparticles were observable inside both the phloem and xylem tissue through HRTEM image of the ultramicrotome cut of its leaf after ten days of application. The authors suggested that the uptake was through the stomata and translocated to xylem and phloem [106]. However, in another study from the same group using the same nanoformulations, the HRTEM image of the foliar application on bean after 30 days have shown that the nanoparticles are only observable in the phloem tissue and none in the xylem tissue [51]. Stomatal uptake pathway of chitosan-Zn nanoparticles upon its foliar application on wheat was reported by Deshpande et al., in which the stomatal localization of the Zn was confirmed via FESEM and fluorescence microscopy [27]. Further internalization of the nanoparticulate system was investigated using confocal laser scanning microscopy, where high content of Zn was found in the embryo, aleurone layer and endosperm of the wheat grain.

## 5. Phytoprotection, Cytoprotection and Genoprotection of Chitosan

Due to its antioxidant, biocompatibility, bioadhesion and action against free radicals, chitosan offers an excellent potential to be used as phytoprotective, cytoprotective and genoprotective agent against toxic agrochemicals [23,108]. Agrochemicals have been widely known to cause phytotoxic, cytotoxic and genotoxic effects on the plant, cell and DNA, respectively [109,110]. Therefore, for that purpose, agrochemicals can be loaded into the spherical nanocapsule matrix of chitosan, which in turn formed a protective barrier or shell and prevent the direct contact of the toxic pesticide with the cell or DNA [111,112]. To support this hypothesis, greenhouse and nursery tests have been carried out for phytotoxicity analysis, while in vitro cell viability studies via MTT assays have been conducted for cytotoxicity analysis. The DNA damage test via comet assay has also been employed for genotoxicity analysis.

Enhancement in the growth parameters, including total fresh weight, leaf area, root weight and leaf mass of chilli seeds exposed to the treatment was reported in the following order: control (untreated) < bulk chitosan < chitosan nanoparticles. The non-phytotoxic effect of chitosan, and the ability of its nano-sized particles to further extend the growth of the seedlings was indicated [48]. The findings were also supported in the supplementation of chitosan nanoparticles on wheat and barley plants [113]. In another study, chitosan-thiamine nanoparticles exhibited a significant reduction in the cell death of the *F. oxysporum*-infected roots compared to the untreated ones, hence suggesting the non-cytotoxicity of chitosan-thiamine nanoparticles on the plant cell [40]. Besides, chitosan-alginate and chitosan-tripolyphosphate were utilized as a nanocarrier of herbicides (imazapic and imazapyr), and the cytotoxicity analysis on the onion root indicated that the encapsulation of herbicides could reduce the cell alteration damage compared to free herbicides by 100%. On the other hand, the comet assay showed that the relative damage of the DNA of the hamster ovary cell line exposed to the nanoparticles was significantly reduced compared to the DNA exposed to the free herbicides. Moreover, chitosan-tripolyphosphate-herbicides nanoparticles exhibit the same value as the control (untreated cells), hence, revealing their non-genotoxic effect [67].

Chitosan-hexaconazole nanoparticles could also reduce the cytotoxic effect on the Vero cell line compared to the conventional hexaconazole and free hexaconazole [62]. In addition, the cytotoxicity assessment on the human lens epithelial cell line exposed to chitosan-carbendazim nanoparticles revealed its lower toxic effect compared to the free carbendazim. Chitosan nanoparticles were found to be non-toxic to a human lens epithelial cell line [114]. Moreover, conventional and free pyraclostrobin was found to give an acute toxic effect on 24 h exposure against zebrafish, while a significant reduction in LC_50_ was observed in the exposure of the encapsulation of pyraclostrobin in chitosan-poly(2-dimethylamino-ethyl methacrylate) microcapsule against zebrafish [115], therefore, providing evidence of the potential of chitosan as a phytoprotective, cytoprotective and genoprotective agent against toxic pesticides. Apart from that, in the field of pharmaceuticals, nanocapsules and nanoemulsions of chitosan loaded with triclabendazole have been reported to be able to significantly lower the cytotoxic effects on intestinal absorptive cells compared to their counterparts [116]. In another report, chitosan-quinapyramine sulfate nanoparticles exerted the ability to lower the cytotoxic and genotoxic effects on the HeLa cell line compared to their conventional counterparts [117]. The nanoformulations were also reported as being able to enhance the efficiency of the drug in the treatment of trypanosomes and prolong the survivability of infected rabbits.

## 6. Agronanochemicals Exert Negative Impacts on Human Health and Environment Wellbeing

Reducing the negative impacts of agrochemicals on human health and the environment has become a primary concern among researchers due to the unavoidable use of agrochemicals in crop management. The risk or hazards to humans depends on the toxicity of the agrochemicals used and the amount and form of exposure experienced via an application or residues in food and drinking water [118]. Moreover, the use of conventional agrochemicals causes significant adverse impacts on the environment and are a threat to both terrestrial and aquatic life, as they are dissipated and leached. Improper handling and prolonged exposure of agrochemicals in the agricultural industry results in unwanted consequences such as skin and eye irritation, nausea, headaches, vomiting and shortness of breath [119,120].

As discussed earlier, the encapsulation of agrochemicals in nanoparticles of chitosan offers a controlled release behavior, which in turn helps to decrease the wastage and leaching of the agrochemicals. The long circulation time and high efficiency of chitosan-based agronanochemicals also can reduce the application dosage of the active ingredient, thus, minimizing the environmental issues such as run-off and accumulation of agrochemicals. Moreover, studies of soil microbial populations and their activities upon application of agrochemicals provide an understanding of the elemental cycles in the soil, including the enzymatic activities and structures of the microbial population of bacteria, yeast, algae, protozoa, actinomycetes and fungi [121,122]. With these in mind, the effect of chitosan-based agronanochemicals compared to their counterpart free agrochemicals on the soil microbial population, has been evaluated. Namasivayam et al. reported a drastic reduction in soil enzyme activity and microbial application upon the application of free herbicide (paraquat), while the application of chitosan-herbicide nanoparticles showed no significant effect compared to the control [68]. In another work, Maruyama et al. reported the improved effect on the soil microbial population at seven days of the application of chitosan-alginate-herbicides nanoparticles compared to their free herbicides (imazapic and imazapyr) [67]. Thus, these findings highlight the ability of chitosan nanocarriers to minimize the adverse side effect of the toxic agrochemicals on soil health.

## 7. Future Perspectives

Even though there is a lot of successful works reported in the potential of chitosan-based agronanochemicals in plant cultivation and disease management, it is still early to form conclusions from the results since data from nursery and greenhouse (in vivo) studies is still lacking, let alone from real field evaluations. The effectiveness of chitosan-based agronanochemicals in comparison to conventional agrochemicals to tackle the real problems faced by the agricultural industry should be evaluated. In addition, the beneficial effect of chitosan nanoparticles in pest and disease management in crops depends on multiple factors; particle size, exposure concentration, solubility, biodegradability, surface charge and their ability to permeate and penetrate the cell wall of the “enemy”. Hence, further holistic evaluations of the effect of these factors as disease-suppressing agents in crops are needed to enable the modulation of chitosan-based agronanochemicals with the desired properties. Moreover, more data is needed to determine the actual movement mechanism of chitosan-based agronanochemicals in a plant, where the uptake, translocation and transportation of the chitosan-based agronanochemicals might rely on the particle size, morphology, surface charge, solubility, bioavailability, plant types and their effective exposure concentrations.

## 8. Conclusions

Chitosan by itself can act as a growth promoter as well as provide antimicrobial action, enhance plant immunity and defenses against the plant pathogens. Alternatively, agrochemical active ingredients can be loaded or encapsulated into chitosan nanoparticles for the formation of a potent biocide, in which the resulting non-toxic and biocompatible chitosan nanoparticles can act as a protective wall, and subsequently shield plants from the toxic effects of the agrochemicals loaded into them. Also, the chitosan-based agronanochemicals aim to enhance the efficient delivery of active agents to the target site, able to sustain it for a long time and consequently improve the agrochemicals’ efficacy. Moreover, the formulated systems could enhance uptake, minimizing leaching and runoff of agrochemicals that can cause health and environmental concerns. Therefore, chitosan-based agronanochemicals can provide a sustainable alternative to conventional agrochemicals in crop disease management.

## Figures and Tables

**Table 1:** Some recent works on the use of chitosan nanoformulations as a plant growth promoters.

Nanoformulations, Molecular Weight (MW), Deacetylation Degree and Final pH of the Product	Plant and Application Type	Average Size * and Zeta Potential	Findings	Ref.
Nano-chitosan, 600 kDa, 85%, pH 6.0	Robusta coffee (*Coffea canephora*), foliar spray	420, 750 and 970 nm ^c^	Increase chlorophyll content (30–50%), enhance nutrient uptake (10–27% N, 17–30% P, 30–45% K) and photosynthesis rate (30%).	[34]
Nano-chitosan, 110 kDa, 85%–90%, pH 4.0	Chilli (*Capsicum annuum*), seed treatment	163 nm ^a^, +60.4 mV	Enhance in total root and leaf fresh mass up to 77% and 28%, respectively upon application of 1 mg/L of nano-chitosan. The increase of leaf catalase (33%) and peroxidase activities (23%) also been observed.	[48]
Nano-chitosan, 100–399 kDa,	Bean (*Phaseolus vulgaris*), seed treatment	46 nm ^a^	Promote seed germination (123% after 72 h) and radical length (231% after 72 h) under salinity stress.	[35]
Nano-chitosan, pH 7.0–9.0	Maize (*Zea mays*), seed treatment	80–100 nm ^d^	Promote seed germination (37%), plant height (1.5-fold increase) and leaf area (2-fold increase).	[49]
Nano-chitosan, pH 4.8	Chickpea (*Cicer arietinum*), seed treatment	10–30 nm ^b^, −37 mV	Enhance germination (100%), seedling vigor index (57%) and vegetative biomass of seedlings (3-fold).	[43]
Chitosan-polymethacrylic acid-NPK nanoparticles	Wheat (*Triticumaestivum*), foliar spray	26 and 31 nm ^b^	Enhance harvest index (24%), crop yield (59%), and mobilization index (42%).	[36]
		20 nm ^b^	Enhance polysaccharides (10%) and total saccharides (11%).	[50]
	French bean (*Phaseolus vulgaris*), foliar spray	20 nm ^b^	Enhance plant growth, nutrient uptake, and biomass accumulation. The nanoformulations was found on the leaf phloem via HRTEM image	[51]
	Pea (*Pisum sativum*), seed treatment	20 nm ^b^	Induce mitotic cell division (1.5 fold) and enhance of total soluble protein (i.e., legumin β, vicilin 1, 2 and 3, and convicilin)	[52]
Chitosan-Cu nanoparticles, low MW, 80%	Maize (*Surya local*), seed treatment	150 nm ^b^, +22.6 mV	Increase α-amylase and protease activity as well as promote seedling growth.	[37]
Chitosan-Cu nanoparticles, 50–190 kDa, 80%	Maize (*Zea mays*), foliar spray	361 nm ^a^,+22.1 mV	pH-responsive sustained release of Cu was obtained. Promote seedling growth (significant increase in plant height, stem diameter, and root length).	[26]
Chitosan-Zn nanoparticles, 60 kDa, 85%	Wheat (*Triticum durum*), foliar spray	325 nm ^a^, +42.3 mV	Stomatal localization of nanoparticles was observed. Increase grain zinc content by up to 42%.	[27]
Chitosan-γ-polyglutamic acid-gibberellic acid nanoparticles, 290 kDa, 75%–85%, pH 4.5	French bean (*Phaseolus vulgaris*), seed treatment	134 nm ^a^, −29.0 mV	61% of the encapsulation efficiency of hormone in the nanoformulation. Offer sustained-release with 58% after 48 h. Exhibited high biological activity with 50–70% enhance of seed germination, leaf area, and root development compared to counterpart.	[39]
Chitosan-gibberellic acid nanoparticles, 27 kDa, 75%–85%, pH 4.5	French bean (*Phaseolus vulgaris*), seed treatment	450 nm ^a^, +27.0 mV	90% of the encapsulation efficiency of hormone in the nanoformulation. Offer stability up to 60 days with pH and temperature-controlled release mechanism. Upon treatment, the seedlings showed an increase of leaf area, chlorophyll and carotenoids amount.	[38]
Chitosan-thiamine nanoparticles, 27 kDa, 85%	Chickpea (*Cicer arietinum*), seed treatment	596 nm ^a^, +37.7 mV	99% of the encapsulation efficiency of hormone in the nanoformulation. Enhance seeds germination and induce more defense enzymes (peroxidase, glucanase, chitinase, polyphenol oxidase, protease, and chitosanase activity) and increase 10-fold auxins level compared to the untreated seeds.	[40]

* ^a^ hydrodynamic size, ^b^ high-resolution transmission electron microscopy (HRTEM) diameter size, ^c^ field emission electron microscopy (FESEM) diameter size and ^d^ unmentioned.

**Table 2:** Some of the recent works on the use of chitosan nanoformulations as sustainable alternatives to conventional agrochemicals.

Plant Pathogen	Nanoformulations, Average Size *, Zeta Potential and its Application	In Vitro/In Vivo	Findings	Ref.
***Alternaria solani, Fusarium oxysporum*, and *Pyricularia grisea,***	Nano-CS, 10-30 nm ^b^, –37 mV (fungicides)	In vitro	High inhibition on mycelial growth with the percentage of inhibition rate recorded at 92%, 87%, and 72% for *P. grisea, F.* *oxysporum* and *A. solani*, respectively.	[43]
***Aphis gossypii***	CS-polyacrylic acid nanoparticles, 50 nm ^a^ (insecticides)	In vivo, reared on castor leaves	The mean number of eggs/females reduce significantly under the laboratory conditions and field conditions with 76% and 61%, respectively.	[41]
***Callosobruchus chinensis***		In vivo, reared on castor leaves	The mean number of eggs/females reduce significantly under the laboratory conditions and store conditions with 74% and 70%, respectively.	[41]
***Callosobruchus maculatus:***		In vivo, reared on soybean	The mean number of eggs/females reduce significantly under the laboratory condition and store condition with 84% and 74%, respectively.	[41]
***Colletotrichum*** ***Gloeosporioides* and *Alternaria* spp.**	Nano-CS, 406 nm ^a^, –4.9 to –7.9 mV (fungicides)	In vitro	Higher inhibition on mycelial (up to 82%) and sporulation of fungus, compared to the counterpart. Enhance seeds germination.	[44]
***Curvularia lunata***	CS-Cu nanoparticles, 361 nm ^a^, +22.1 mV (fungicides)	In vitro and In vivo (Maize, *Zea mays*)	Induce more defense response: 1.5–2 fold of peroxidase, a significant amount of superoxide dismutase, 2–3 fold of phenylalanine ammonia-lyase, and a significant amount of polyphenol oxidase.	[26]
***Fusarium*** ***oxysporum***	CS-CuO, 350 nm ^b^, –26.8 mV; CS-ZnO, 441 nm ^b^, –24.5 mV; and CS-Ag, 348 nm ^b^, –49.1 mV (fungicides)	In vitro and In vivo (chickpea, *Cicer arietinum*)	In vitro results showed that the antifungal activity follows: CS-ZnO > CS-CuO > CS-Ag, while in vivo results showed that the wilt disease reduction follows: CS-CuO (47%) > CS-ZnO (40%) > CS-Ag (33%).	[53]
***Fusarium graminearum***	Nano-CS, 181 nm ^a^, +45.6 mV (fungicides)	In vitro and in vivo (wheat)	85% inhibition of mycelial growth in plate treated with 5000 mg/mL of CS nanoparticles (in vitro) and 53% reduction in disease severity on wheat (in vivo). Deformation and dehydration of fungus mycelial growth also can be seen.	[54]
	Nano-CS, [1] 181 nm ^a^, +45.6 mV; [2] 310 nm^a^, +33.2 mV; [3] 340 nm ^a^, +21.7 mV (fungicides)	In vitro and in vivo (wheat)	Inhibition rate (%) at 1000 mg/mL follows: (1) Nano-CS (71.1%) > (3) Nano-CS (17.7%) > (2) Nano-CS (14.1%)	[45]
	CS-Cu nanoparticles, 220 nm ^a^, +40.0 mV (fungicides)	In vitro	Minimum inhibitory concentration after one week incubation follows: Cu (250 µg/mL) > CS-Cu nanoparticles (17.5 mg/mL) > chitosan (10 mg/mL).	[55]
***Fusarium verticillioids***	CS-Cu nanoparticles, 296 nm ^a^, +19.6 mV (fungicides)	In vivo (Maize, *Zea mays*)	At 4 and 8 h after treatment, the disease has been reduced by 48% and 50%, respectively.	[56]
***Pyricularia grisea***	Nano-CS, 83 nm ^a^, –28.0 mV (fungicides)	In vitro and In vivo (rice, *Oryza sativa*)	No inhibitory activity was observed in the in vitro. However, in vivo results revealed its ability in suppressing the disease with zero percent disease incidence at 10 days after infection, where 100% disease incidence was observed in control.	[57]
		In vitro and In vivo (finger millet, *Eleusine coracana*)	In the in vitro evaluation, 65% of radial growth inhibition was obtained. Meanwhile, delayed disease symptom (25 days) and low disease infection (23%) was observed in the in vivo evaluation, while for control, the symptoms started appear in 15 days and 100% disease infection was recorded. Enhance in peroxidase activity level (reached maximum on day 50) also been observed.	[58]
	CS-Cu nanoparticles, 88 nm ^a^, –29.0 mV (fungicides)	In vitro and In vivo (finger millet, *Eleusine* *coracana*)	Induce resistance against the pathogen attack: a 2-fold increase in chitinase and chitosanase and produce more protease inhibitors, peroxidase, β-1,3 glucanase, and polyphenol oxidase compared to the untreated plant.	[59]
***Pyricularia oryzae***	Nano-CS, 28 nm ^b^, +49.0 to +53.0 mV and CS-protocatechuic acid, 33 nm ^b^, +11.0 mV (fungicides)	In vitro	The diameter of inhibition zone follows: CS-protocatechuic acid nanoparticles > protocatechuic acid > chitosan nanoparticles. Up to a 3-fold increase of the inhibition zone compared to the counterpart.	[60]
***Verticillium dahliae***	Nano-oleoyl-CS, 297 nm ^c^ (fungicides)	In vitro	The nanoparticles internalized the fungal cell, hence leads to the deformation of spore and hyphae, thickened cell walls, cease of organelles and cytoplasmic vacuolation.	[61]

* ^a^ hydrodynamic mean size, ^b^ high-resolution transmission electron microscopy (HRTEM) mean diameter size and ^c^ field emission electron microscopy (FESEM) diameter size.

**Table 3:** Some of the recent works on the use of chitosan (CS) nanocarriers for existing agrochemicals as the active ingredient (AI.).

Agrochemicals Type and Its Active Ingredient	Nanocarrier Formulations, Loading Content % (LC), Loading Efficiency % (LE), Encapsulation Efficiency % (EE), and its Average Size *	Plant Pathogen	In Vitro/In Vivo	Findings	Ref.
Fungicide, Dazomet	CS nanoparticles, [1] 276 nm ^b^, 28% (LC), 78% (EE); [2] 32 nm ^b^, 48% (LC), 98% (EE); [3] 31 nm ^b^, 35% (LC), 85% (EE); [4] 7 nm ^b^, 33% (LC), 83% (EE)	*Ganoderma boninense*	In vitro	Controlled release with saturation release of 97.9% and half release time (t_1/2_) of 11 h at pH 5.5. Increase fungicidal activity up to 30-fold compared to their counterparts.	[29]
Fungicides, Hexaconazole and Dazomet	CS nanoparticles, [1] 157 nm ^b^, 17% (LC), 67% (EE); [2] 58 nm ^b^, 17% (LC), 67% (EE); [3] 31 nm ^b^, 17% (LC), 67% (EE); [4] 5 nm ^b^, 13% (LC), 64% (EE)	*Ganoderma boninense*	In vitro	Controlled release with half release time (t_1/2_) up to 66 and 19 h for hexaconazole and dazomet, respectively, at pH 5.5. Increase fungicidal activity up to 40-fold compared to their counterparts.	[30]
Fungicide, Hexaconazole	CS nanoparticles, 100 nm ^b^, 73% (EE)	*Rhizoctonia solani*	In vitro	Controlled release with prolongs the release time of hexaconazole up to 14 days at pH 8.3 while the conventional pesticides only last up to 5 days. Significant higher antifungal activity compared to the conventional counterpart.	[62]
Fungicide, Hexaconazole	CS nanoparticles, [1] 272 nm ^b^, 11% (LC), 56% (EE); [2] 169 nm ^b^, 17% (LC), 67% (EE); [3] 32 nm ^b^, 15% (LC), 65% (EE); [4] 18 nm ^b^, 15% (LC), 65% (EE)	*Ganoderma boninense*	In vitro	Controlled release with saturation release of 99.9% and half release time (t_1/2_) of 42 h at pH 5.5. Increase fungicidal activity up to 3-fold compared to their counterparts.	[28]
Fungicide, Pyraclostrobin	CS-lactide nanoparticles, [1] 128 nm ^a^, 18% (LC), 45% (EE); [2] 90 nm ^a^, 11% (LC), 85% (EE); [3] 77 nm ^a^, 2% (LC), 91% (EE);	*Colletotrichum* *gossypii*	In vitro	Better stability of AI under light stress with 81% compared to the counterpart with 41%. Controlled release (75%) of AI up to 10 h at pH 8.3. High fungicidal activity with up to 85% inhibition rate at day 7 of incubation.	[63]
Fungicide, Pyraclostrobin	Quarternized CS-silica nanoparticles, 110 nm ^b^, 27%–42% (LC)	*Phomopsis asparagi*	In vitro	Controlled release (72%) with prolongs release time up to 13 h. Inhibition percentage of fungi up to 95%	[46]
Fungicides, Tricyclazole and Hexaconazole	CS-Ag nanoparticles, 17 nm ^b^	*Pyricularia oryzae*	In vitro	Significantly increased the inhibition zone by 2-fold compared to the counterpart	[64]
Fungicide, Avermectin	CS-lanthanum-nanoparticles, 333 nm ^a^, 46% (LE), 65% (EE)	*Magnaporthe grisea*	In vitro and In vivo	Rapid release on the first 36 h followed by sustained release until day-10. No inhibitory of fungus was observed in the in vitro study. However, significant disease reduction was observed in the in vivo study (Rice, *Oryza sativa*).	[65]
Fungicide, Tebuconazole	CS-porphyrinic-pectin nanoparticles, 100 nm ^c^, 30% (LE)	*Xanthomonas campestris, Pseudomonas syringae,* and *Alternaria alternate*	In vitro	Metal-organic frameworks (MOFs) capsule comprise of chitosan, porous porhpyrinic, and pectin demonstrated a stimuli-responsive sustained release of AI with prolonged-release time up to 174 h at pH 7. The nanocapsule exhibited high antimicrobials activities and no phytotoxic effect on Chinese cabbage.	[66]
Herbicides, Imazapic, and Imazapyr	CS-alginate nanoparticles, 378 nm ^a^, 62% (EE) of imazapic, 71% (EE) of imazapyr;CS-tripolyphosphate nanoparticles, 479 nm ^a^, 59% (EE) of imazapic, 70% (EE) of imazapyr	*Bidens pilosa*	In vivo	After 300 min under gentle agitation, 30% (imazapic) and 20% (imazapyr) were released in CS-alginate nanoparticles, while 59% (imazapic) and 9% (imazapyr) were released in CS-tripolyphosphate nanoparticles. Meanwhile, free imazapic and imazapyr were released up to 55% and 97%, respectively, hence, showing the slow-release formulation of the nanoparticulate system. The encapsulation of herbicides also reduced the toxicity of herbicides against the nontarget organism while maintaining its herbicidal activity on the tested weeds.	[67]
Herbicide, Paraquat	CS-Ag nanoparticles, 100 nm ^c^, 90% (EE)	*Eichhornia crassipes*	In vivo	Improved herbicidal activity on the tested weed with a 90% release of paraquat was observed for up to 24 h. Improved the microbial population, bacteria, and yeast compared to its free herbicide.	[68]
Nematicide, Avermectin	CS-γ-polyglutamic acid nanoparticles, 61 and 56 nm ^b^, 31% (LC), 35% (EE)	*Caenorhabditis elegans*	In vitro	The controlled release rate governed by pH. The mortality rate of nematodes was significantly increased by 29%, compared to its counterpart.	[69]

*^,a^ hydrodynamic mean size, ^b^ high-resolution transmission electron microscopy (HRTEM) mean diameter size and ^c^ field emission electron microscopy (FESEM) diameter size.
